# A Sedentary Behaviour Reduction Programme in Patients With Peripheral Arterial Disease: A Mixed‐Method Feasibility Study

**DOI:** 10.1155/ijvm/1159242

**Published:** 2026-04-14

**Authors:** Marwa Said, Wael Tawfick, Charlotte L. Edwardson, Marcia Carvalho, Sherif Sultan, Mahmoud Alawy, Eleftheria Filandrianou, Megan Nolan, Jennifer Jones

**Affiliations:** ^1^ School of Medicine, University of Galway, Galway, Ireland, universityofgalway.ie; ^2^ National Institute for Prevention and Cardiovascular Health, Galway, Ireland; ^3^ Diabetes Research Centre, College of Life Sciences, University of Leicester, Leicester, UK, le.ac.uk; ^4^ Leicester Diabetes Centre, Leicester General Hospital, University Hospitals of Leicester NHS Trust, Leicester, UK, nhs.uk; ^5^ Department of Vascular Surgery, Galway University Hospital, Galway, Ireland, saolta.ie; ^6^ Integrated Care Wexford, Chronic Disease Hub, Enniscorthy Primary Care Centre, Enniscorthy, Co Wexford, Ireland

**Keywords:** activPAL, digital health, feasibility study, peripheral arterial disease (PAD), physical activity, sedentary, sitting, wearable technology

## Abstract

**Background:**

People with peripheral arterial disease (PAD) have high levels of sedentary behaviour (SB), which contributes to declining mobility, poorer quality of life and increased cardiovascular risk; however, few studies have targeted reducing SB in this population.

**Objective:**

The study is aimed at evaluating the feasibility and acceptability of delivering and evaluating a 12‐week remotely delivered intervention designed to reduce sedentary time in people with PAD.

**Methods:**

This was a single‐arm, single‐centre feasibility study in participants with PAD. The intervention combined online education, a wearable physical activity tracker and weekly coaching calls. At baseline and 12 weeks, SB and physical activity were measured with activPAL, functional capacity was assessed remotely using the Timed Walk app for the 6‐min walk test and semistructured interviews were conducted at 12 weeks to evaluate acceptability.

**Results:**

Thirty participants provided consent (77% recruitment rate) and took part in the study, with 21 (70%) attending follow‐up. Valid activPAL data at baseline and follow‐up was provided by 18 participants (60% of those consented). At baseline, participants spent 63% of their waking day sitting (9.58 h/day), which was slightly lower at 12 weeks (60%). Qualitative analysis identified four themes covering awareness, motivation, engagement and barriers. Participants valued weekly calls and feedback on physical activity from the wearable.

**Conclusion:**

Overall, the intervention was feasible and acceptable, and data suggest that it may reduce sedentary time. Methods to enhance retention and compliance with the activPAL would be needed for a larger trial.

**Trial Registration:**

ClinicalTrials.gov identifier: NCT05961943

## 1. Introduction

Peripheral arterial disease (PAD) is a common yet often underrecognised condition, affecting more than 200 million people worldwide [1]. It is characterised by atherosclerotic narrowing of the lower limb arteries, leading to reduced blood flow and an increased risk of serious cardiovascular events such as stroke and myocardial infarction [2]. An ankle‐brachial index (ABI) of ≤ 0.90 is associated with more than a twofold increase in the 10‐year risk of coronary events, cardiovascular mortality and all‐cause mortality. Within 5 years, 20% of patients with intermittent claudication (IC) will experience a myocardial infarction or stroke with a mortality rate of 10%–15% [2]. Multiple risk factors contribute to PAD [1], and growing evidence highlights the importance of lifestyle factors, particularly regular physical activity and low levels of sedentary behaviour, in its management [3].

Sedentary behaviour is defined as waking time spent in sitting, lying or reclining positions with low energy expenditure (1.0–1.5 times the basal metabolic rate) [4]. The World Health Organization (WHO) recommends that adults undertake at least 150–300 min of moderate‐intensity aerobic activity per week (or an equivalent amount of vigorous activity) and acknowledges the importance of limiting sedentary time, although it does not specify absolute limits [5]. Evidence with accelerometry‐based measurements indicates that accumulating 9.5 h or more per day of sedentary time is strongly associated with an increased risk of all‐cause mortality [6].

High levels of sedentary behaviour have been linked to negative cardiovascular outcomes, exacerbating PAD symptoms and further limiting mobility [6]. Conversely, regular physical activity is known to improve blood flow, reduce symptoms of IC and improve quality of life for individuals with PAD [7]. Despite this, the impact of reducing sedentary behaviour has not been adequately studied in people with PAD, with most research focusing on increasing moderate to vigorous physical activity rather than addressing sedentary behaviour directly. For patients with PAD, reducing sedentary time may have the potential to mitigate the progression of the disease and improve overall function [3].

Interventions targeting sedentary behaviour are gaining attention in other chronic conditions, with promising results in populations with diabetes, obesity and cardiovascular disease [8, 9]. For people with PAD, interventions that replace sedentary time with light or moderate‐to‐vigorous activity may be especially beneficial, given pain and mobility challenges. Educational support, personalised goals and tracking tools could help promote active living while limiting sedentary time.

### 1.1. Aim

The study is aimed at evaluating the feasibility and acceptability of delivering and evaluating a 12‐week remotely delivered intervention designed to reduce sedentary time in people with PAD.

## 2. Objectives

### 2.1. Primary Objectives

The primary objectives of the study are as follows: to evaluate key aspects of feasibility, including recruitment, attrition and data completion rates, to determine the practicality of the intervention, and additionally, to assess the acceptability of the intervention and gather insights into participants’ experiences with the intervention used.

### 2.2. Secondary Objectives

The secondary objectives are as follows: to evaluate potential changes in sedentary behaviour and physical activity levels using research‐grade accelerometry‐derived data (activPAL) and, additionally, to assess potential changes in 6‐min walk test (6MWT) performance using the Timed Walk application.

## 3. Materials and Methods

### 3.1. Study Design

A mixed‐methods single‐group, pre–post, feasibility study in individuals with PAD. The intervention consisted of a remotely delivered online education programme, supplemented by weekly coaching calls. A consumer physical activity tracker (Huawei Band 6) was used to deliver vibration prompts, reminding participants to break up sitting after 60 min of inactivity. Baseline and 12‐week measurements were collected to assess recruitment, retention, acceptability, participant experiences and data completion rates.

### 3.2. Participants

#### 3.2.1. Study Setting

Recruitment took place at the vascular outpatient clinic at Galway University Hospital (GUH), Ireland. The Croí Heart and Stroke Centre was used to meet with some participants for baseline assessment and follow‐up visits when this was more feasible for them.

#### 3.2.2. Participants and Sample Size

Symptomatic PAD patients attending routine appointments at the GUH vascular outpatient clinic were screened, and those meeting the inclusion criteria were invited to participate. The study was planned as a pilot feasibility study, with recruitment taking place over a 6‐month period from the vascular outpatient clinic. Following screening and eligibility assessment, 30 participants were successfully enrolled. This sample size aligns with pilot study recommendations (24–50 participants) [10] and was considered appropriate given the limited research on reducing sedentary time in patients with PAD.

#### 3.2.3. Inclusion Criteria

Participants were eligible if they were adults (≥ 18 years) with established PAD, indicated by an ABI of < 0.90 in at least one lower extremity, a toe‐brachial index of < 0.60 or arterial occlusive disease confirmed by duplex ultrasonography, CT angiography or MRI.

#### 3.2.4. Exclusion Criteria

Participants were excluded if they had severe impairments affecting mobility or cognition as documented in their medical records, significant comorbidities that hindered their ability to participate in physical activity or implantable cardioverter‐defibrillators (ICDs) or pacemakers. Additionally, pregnant or lactating women were not eligible to participate in the study. As the intervention included an education session in English, potential participants with a poor command of the English language were excluded.

### 3.3. Recruitment Process

**Figure 1:**
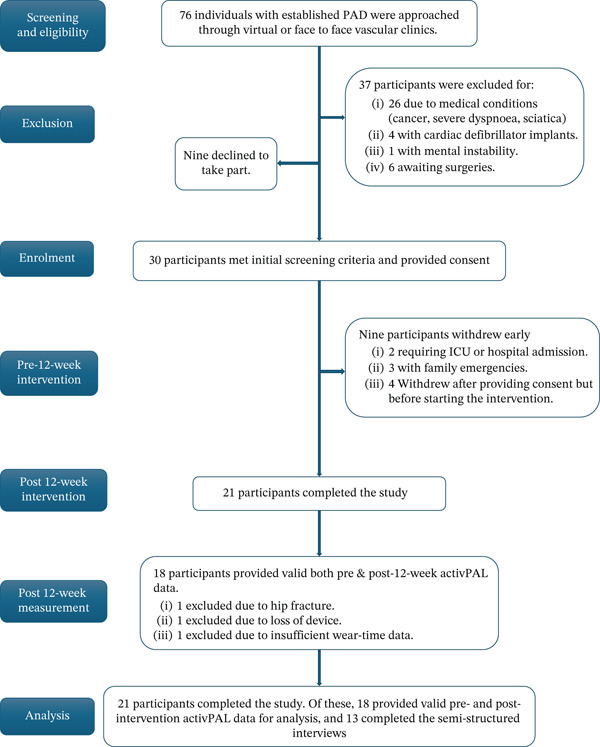
Flowchart of study enrolment process and analysis.

Recruitment was conducted at GUH’s vascular outpatient clinics. The vascular team screened patients based on the inclusion criteria. Patients expressing interest were given a patient information leaflet and the opportunity to ask questions. Those who agreed to participate signed a consent form (Figure 1).

### 3.4. Procedures

Participants who provided consent were invited to attend a baseline assessment, either in person at the Croí Heart and Stroke Centre or remotely via telephone or Zoom. During the initial assessment, demographic data were recorded using a case record form, which captured baseline characteristics such as date of birth, gender, smoking status (current, former and never), ABI, medical history and health‐related conditions, including Type 2 diabetes, heart disease, hypertension, dyslipidemia, cardiac, functional impairment and respiratory conditions following the Society for Vascular Surgery (SVS) reporting standards [11].

All participants were provided with an activPAL3 accelerometer‐based device (PAL Technologies, Glasgow, United Kingdom), either during their in‐person appointment or via a postal pack, along with instructions on how to secure the device to the thigh and wear it continuously 24 h per day for 7 consecutive days. The researcher had follow‐up contact with participants to ensure that the device was properly fitted, functioning correctly, and that participants were completing daily diary entries on the provided log sheet.

During the initial assessment, participants completed the 6MWT using the Timed Walk application (Dario Salvi, Malmö University) [12]. This remote application has been validated as a reliable and feasible tool for assessing 6‐min walk distance [13]. Participants underwent all measurements at baseline and 12 weeks from recruitment. Semistructured interviews were also conducted at the 12‐week follow‐up to discuss participants’ perceptions and feedback regarding the programme’s acceptability and suitability and to suggest any potential improvements for future delivery.

### 3.5. Patient and Public Involvement (PPI) Group

The PPI group from the Croí Heart and Stroke Centre played a key role in reviewing the programme and providing feedback. This established group carefully evaluated all aspects of the research methodology to ensure it was practical and participant‐friendly. While no changes were ultimately required, their review confirmed that the study design, procedures and materials were appropriate and well‐suited to the study’s objectives.

The PPI group was provided with several key components of the programme for review, including the following:

• An online education session adapted from the successful SMART Work and Life intervention, which highlighted the adverse effects of sedentary behaviour and introduced strategies to reduce sitting time [14, 15].
• Tools for assessing and self‐monitoring daily sedentary time, along with guidance on using various free apps to track activity levels.
• An action plan and goal‐setting worksheet designed to encourage participants to reduce sedentary time to less than 50% of waking hours and incorporate movement every 30 min.

In addition to these elements, the group was briefed on the planned use of a wrist‐worn activity tracker (Huawei Band 6) and weekly phone or video coaching sessions to provide ongoing support and motivation. Their feedback confirmed that these components were well‐structured and aligned with the study’s aims.

### 3.6. Intervention

Developing effective sedentary behaviour reduction interventions necessitates an understanding of what drives behavioural change and the underlying mechanisms influencing sedentary time. The ‘Behaviour Change Wheel’ framework identifies nine key intervention functions, including education, persuasion and training, to facilitate behavioural modifications [16, 17].

This study carefully selected behaviour change techniques (BCTs) to form an intervention aimed at reducing sedentary time in patients with PAD. The selection process considered factors such as affordability, practicability and acceptability to ensure feasibility within the target population. Table 1 outlines the BCTs incorporated into the intervention, which consists of an online education programme, health coaching calls and a physical activity tracker [16, 18].

**Table 1:** Mapping of behaviour change techniques (BCTs) utilised in the intervention‐to‐intervention components.

BCT	Description	Education programme	Coaching calls	Activity tracker
Goal setting 1.1	Establishing a specific, measurable target for behaviour change.	✓	✓	—
Problem solving 1.2	Identifying barriers to behaviour change and developing strategies to overcome them.	✓	✓	—
Action planning 1.4	Creating a detailed plan that specifies when, where and how to perform the desired behaviour.	✓	✓	—
Review behaviour goals 1.5	Regularly assessing progress towards behaviour goals.	—	✓	—
Discrepancy between current behaviour and goal 1.6	Highlighting the difference between current behaviour and the desired goal to motivate change.	✓	✓	—
Commitment 1.9	Encouraging a promise to change behaviour.	—	✓	
Feedback on behaviour 2.2	Providing information on how well someone is performing the desired behaviour.	—	✓	✓
Self‐monitoring of behaviour 2.3	Tracking one’s own behaviour over time to increase awareness and control.	—	✓	✓
Information about antecedents 4.2	Providing information on triggers that lead to the behaviour.	✓	—	—
Information about health consequences 5.1	Explaining the health impacts of the behaviour.	✓	—	—
Prompts/cues 7.1	Using reminders or signals to encourage the behaviour.	—	—	✓
Habit formation 8.3	Repeating a behaviour in a consistent context to make it habitual.	✓	✓	—
Credible source 9.1	Presenting information from a trustworthy and respected source.	✓	—	—
Pros and cons 9.2	Discussing the advantages and disadvantages of changing the behaviour.	✓	✓	—

#### 3.6.1. Online Education Session

All participants received access to an interactive online education session from the SMART Work and Life programme, which was well received [19] and successfully reduced sitting time in desk‐based workers [14]. It comprised 19 sections, covering topics such as the risks of excessive sitting, benefits of breaking up sitting time, self‐reflection, self‐monitoring, use of prompts, goal setting, action planning and overcoming personal barriers. Participants could complete it at their own pace, typically taking 60–90 min. The session emphasised reducing sitting, aiming for less than 50% of waking hours spent sitting and incorporating movement every 30 min.

#### 3.6.2. Self‐Monitoring and Prompts

To support behaviour change, participants were given a wrist‐worn physical activity consumer device (Huawei Band 6) to encourage them to reduce sitting time and break up prolonged sedentary time. This device provided real‐time feedback on daily activity levels, helping participants monitor their progress. In addition, it delivered a vibration prompt after 60 min of inactivity, reminding participants to break up prolonged sitting.

##### 3.6.2.1. Wearable Feedback Standardisation

To ensure consistency across participants, all wrist bands were set up according to a standardised protocol. Vibration prompts and other settings were identical for all participants. Participants received uniform instructions on interpreting alerts and reminders, and coaching calls reinforced correct device use, ensuring comparable behavioural feedback throughout the participants.

#### 3.6.3. Health Coaching Calls

Following the online education session, the researcher provided ongoing support and motivation for participants through weekly phone or video calls via Zoom or Teams. These coaching calls focused on reviewing progress, identifying barriers and setting personalised goals to reduce sedentary behaviour and increase movement. Participants discussed their individual challenges and, together with the researcher, developed strategies to overcome them. Coaching was framed using the COM‐B model to explore participants’ capability, opportunity and motivation to reduce sitting time. Participants were offered 12 weekly calls.

##### 3.6.3.1. Health Coach Training and Fidelity

The health coach received structured training prior to delivering the intervention, which also included an overview of sedentary behaviour research. Training also covered the use of intervention materials, motivational interviewing techniques and guidance on goal‐setting strategies, incorporating participant feedback from activPAL data. The coach practised delivering sessions under supervision and received feedback to ensure consistent delivery. A coaching form was provided for each session to guide content, and all sessions were documented using a session log. This log recorded participant ID, week/date, contact frequency, method, average call length, reasons for missed sessions, topics discussed and any barriers encountered. Fidelity was monitored through periodic review of these logs and random observation of coaching calls to ensure adherence to the protocol and consistency across participants.

## 4. Measurements

### 4.1. Feasibility and Intervention Acceptability

Feasibility was evaluated based on recruitment, retention, intervention adherence and device measurement completion. Thresholds were set based on prior PAD and behavioural intervention studies: recruitment ≥ 60*%* and adherence ≥ 70*%*. Device completion (activPAL) was 60%, below the target, highlighting challenges in sustained use. Semistructured interviews at 12 weeks assessed acceptability, perceived practicality and barriers or facilitators to engagement. These criteria provide a framework for interpreting feasibility and guiding future trials [20].

### 4.2. Measurement of Sedentary Behaviour and Physical Activity

Participants wore the activPAL3 activity monitor on their thigh continuously for 24 h a day, over 7 days, to measure time spent sitting, standing and stepping.

The activPAL was initialised using the manufacturer’s default settings, and data were downloaded using the manufacturer’s software. Events.csv files were generated via PAL Batch and processed using Processing PAL (Version 1.31, University of Leicester, United Kingdom). Processed data were visually inspected through heatmaps. In cases where the sleep–wake classification appeared inaccurate (e.g., early wake and sleep times, late wake and sleep times), the self‐reported log was reviewed, and corrections were applied within Processing PAL if required. Posture‐based outcomes, including total sitting/lying time, prolonged sitting accumulated in bouts of ≥ 30 min and total standing and stepping time, were calculated. Daily stepping time was categorised as light intensity (stepping at a cadence of < 100 steps/min), moderate‐to‐vigorous intensity (stepping at a cadence of > 100 steps/min) and purposeful stepping at a moderate‐to‐vigorous intensity (stepping at a cadence of > 100 steps/min in bouts lasting ≥ 1 min). Additionally, participants maintained a daily diary to log their capturing sleep and wake times and record any instances of device removal.

### 4.3. 6MWT

Functional capacity was assessed remotely at baseline and 12 weeks using the Timed Walk app to conduct the 6MWT [12]. The app delivered standardised instructions, timed the test and recorded the distance covered, enabling measurement of walking capacity at both time points without requiring in‐person assessment. However, the app was temporarily removed from the Google Play Store, rendering it unavailable for Android users during part of the study period.

### 4.4. 12‐Week Follow‐Up Assessments

At the 12‐week follow‐up assessment, participants repeated the baseline measures. Additionally, a semistructured interview at the 12‐week follow‐up gathered feedback on the programme’s acceptability, suitability and potential improvements.

## 5. Outcomes

### 5.1. Primary Outcomes

The study evaluated several key aspects, including recruitment rates of participants with PAD, attrition rates throughout the study and data completion rates for all collected measures. Additionally, participant feedback was gathered to assess their experiences with the intervention and their satisfaction with the intervention components.

### 5.2. Secondary Outcomes

The study explored the potential of the intervention for changing sedentary time and physical activity levels and functional capacity.

## 6. Data Analysis

Data analysis was conducted using IBM SPSS Statistics for Windows, Version 29.0 (IBM Corp., Armonk, New York, United States). Recruitment and retention rates were calculated as the proportion of eligible participants who enrolled in the study and the proportion of enrolled participants who completed follow‐up assessments, respectively. Completion rates for outcome measures were determined by dividing the number of complete datasets by the total number of enrolled participants and multiplying by 100. Follow‐up completion at 12 weeks was similarly calculated as the percentage of participants who completed assessments at that time point out of those expected. Descriptive statistics were used to summarise participant characteristics: Continuous variables were reported as mean and standard deviation (or median and interquartile range, where appropriate) and categorical variables as frequency and percentage. Data distribution was assessed using normality tests.

Baseline and 12‐week follow‐up values were reported for all key outcomes, including sedentary behaviour, physical activity and functional capacity, along with the corresponding observed changes over the intervention period. The potential of the intervention for reducing daily sitting and increasing physical activity was explored using descriptive statistics (mean ± SD, frequency, counts and percentages). In line with good practice recommendations for pilot and feasibility studies [21].

**Figure 2:**
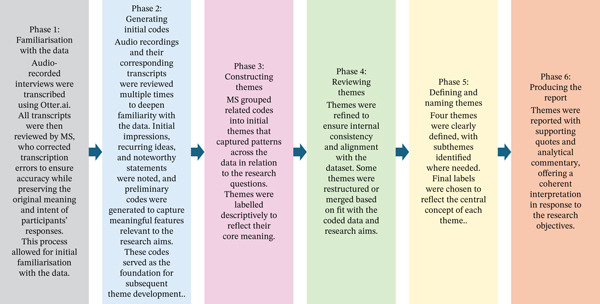
Framework for qualitative data interpretation.

Qualitative interviews were conducted by MS, audio recorded by a digital voice recorder and transcribed using Otter AI (Otter.ai, Inc., Mountain View, California, United States). Of the 21 participants who completed the study, 13 were selected for interview to ensure diversity in age, sex, BMI, ABI and Rutherford classification. Recruitment continued until no new themes emerged, indicating data saturation. All transcripts were independently reviewed by two authors and manually verified before analysis. Reflexive thematic analysis was then conducted, following an iterative and interpretive process of coding and theme development. Figure 2 reveals the framework for qualitative data interpretation.

## 7. Ethics

Ethical approval was obtained from the GUH, Clinical Research Ethics Committee (Approval Number: C.A. 2970). The investigator bore the responsibility of ensuring that no patient underwent any trial‐related examination or activity until they had provided informed consent. The patient had to provide written consent after receiving comprehensive information. The verbal explanation encompassed all aspects outlined in the written material provided to the patient. The investigator apprised the patient of the study’s objectives, methodologies, potential advantages and possible risks, which might have included any discomfort.

Patients were given sufficient opportunity to seek clarification on any matters they found unclear and, if necessary, request further information. Informed consent forms needed to be signed and dated by participants. Additionally, participants were asked for authorisation to share pertinent data with individuals associated with the participating universities or regulatory authorities, where applicable. It was essential to underscore that patients had the freedom to withdraw their consent to participate at any point without facing any penalties or losing benefits to which they would otherwise have been entitled.

## 8. Results

### 8.1. Feasibility and Acceptability

Between September 2024 and February 2025, a total of 76 individuals were approached; 39 were eligible, of whom 30 consented to participate, giving a recruitment rate of 76.9% (≈77%). Follow‐up assessments at 12 weeks were conducted between December 2024 and May 2025. The overall study completion rate was 70.0% (21/30), corresponding to an attrition rate of 30.0%. Attrition reflected participants who withdrew consent or discontinued the study. Of those recruited, 21 (70%) participants provided valid activPAL data at baseline, and 18 (60%) provided valid activPAL data at 12‐week follow‐up, with validity defined as ≥ 3 days of wear. With regard to the weekly health coaching calls, the number completed ranged from 9 to 12 per participant, each lasting 15–30 min. If a participant was unavailable, we attempted to schedule the call on another day. No serious adverse events directly attributable to the intervention or study devices were reported. Table 2 presents the sociodemographic characteristics of all 30 participants, including the 21 who completed the study and the 18 who provided both pre‐ and post‐activPAL measurements.

**Table 2:** Sociodemographic characteristics of the study sample.

	All participants (*n* = 30)	Participants completed the study (*n* = 21)	Participants did not complete the study (*n* = 9)	Differences between groups with *p* values	Participants who provided valid activPAL data (*n* = 18)
Age means (SD) (years)	69.4 (8.0)	71.3 (7.1)	65.0 (8.7)	0.046 ^∗^	70.7 (7.4)
Males *n* (%)	21 (70%)	13 (61.9%)	8 (88.9%)	0.210^#^	11 (61.1%)
BMI median (IQR) (kg/m^2^)	26.5 (25.6–31.1)	27.5 (25.6–32.3)	26.1 (24.1–29.0)	0.651^∞^	27.9 (24.1–32.0)
Alcohol consumption (yes) *n* (%)	13 (43.3%)	8 (38.1%)	5 (55.6%)	0.443^#^	8 (44.4%)
Lowest ABI mean (SD)	0.75 (0.20)	0.71 (0.20)	0.87 (0.19)	0.057 ^∗^	0.71 (0.20)
Lowest TBI mean (SD)	0.49 (0.15)	0.47 (0.16)	0.52 (0.11)	0.500 ^∗^	0.47 (0.17)
Rutherford *n* (%)				0.024*^∞^*	
0 = asymptomatic	0	0	0		0
1 = mild claudication	5 (16.7%)	2 (9.5%)	3 (33.3%)		2 (11.1%)
2 = moderate claudication	13 (43.3%)	8 (38.1%)	5 (55.6%)		6 (33.3%)
3 = severe claudication	12 (40.0%)	11 (52.4%)	1 (11.1%)		10 (55.6%)
Diabetes *n* (%)				0.409^∞^	
0 = none	23 (76.7%)	17 (81%)	6 (66.7%)		15 (83.30%)
1 = not requiring insulin	4 (13.3%)	2 (9.5%)	2 (22.2%)		2 (11.1%)
2 = controlled by insulin	2 (6.7%)	2 (9.5%)	0		1 (5.6%)
3 = Type 1 or uncontrolled	1 (3.3%)	0	1 (11.1%)		0
Smoking *n* (%)				0.063^∞^	
0 = none or remote (> 10 years)	14 (46.7%)	12 (57.1%)	2 (22.2%)		10 (55.6%)
1 = quit 1–10 years ago	6 (20.0%)	4 (19.0%)	2 (22.2%)		3 (16.7%)
2 = current/within last year	10 (33.3%)	5 (23.8%)	5 (55.6%)		5 (27.8%)
HTN				0.595^∞^	
0 = none	8 (26.7%)	7 (33.3%)	1 (11.1%)		6 (33.3%)
1 = controlled with 1 drug	4 (13.3%)	1 (4.8%)	3 (33.3%)		1 (5.6%)
2 = controlled with 2 drugs	17 (56.7%)	13 (61.9%)	4 (44.4%)		11 (61.1%)
3 = requiring > 2 drugs or uncontrolled	1 (3.3%)	0	1 (11.1%)		0
Hyperlipidemia *n* (%)				0.342^∞^	
0 = none	5 (16.7%)	4 (19%)	1 (11.1%)		3 (16.7%)
1 = elevated without drug treatment	0	0	0		0
2 = elevated with dietary treatment	2 (6.7%)	2 (9.5%)	0 (0.0%)		2 (11.1%)
3 = elevated with drug and diet treatment	23 (76.6%)	15 (71.4%)	8 (88.9%)		13 (72.2%)
Cardiac status *n* (%)					
0 = asymptomatic, with normal electrocardiogram	9 (30%)	5 (23.8%)	4 (44.4%)	0.225^∞^	4 (22.2%)
1 = asymptomatic but with remote myocardial infarction by history (6 months) or occult myocardial infarction	11 (36.7%)	8 (38.1%)	3 (33.3%)		6 (33.3%)
2 = any one of the following: stable angina, no angina but significant reversible perfusion defect on dipyridamole thallium scan, significant silent ischaemia (1% of time) on Holter monitoring, ejection fraction 25%–45%, controlled ectopy or asymptomatic arrhythmia or history of congestive heart failure that is now well compensated	10 (33.3%)	8 (38.1%)	2 (22.2%)		8 (44.4%)
Pulmonary *n* (%)				0.435^∞^	
0 = normal	17 (56.7%)	11 (52.4%)	6 (66.7%)		8 (44.4%)
1 = asymptomatic or mild dyspnea	12 (40.0%)	9 (42.9%)	3 (33.3%)		9 (50.0%)
2 = between 1 and 3	1 (3.3%)	1 (4.8%)	0 (0.0%)		1 (5.6%)
3 = vital capacity less than 1.85 L, FEV1 45 mm Hg, supplemental oxygen use medically necessary or pulmonary hypertension	0	0	0		0
Functional *n* (%)					
0 = no impairment	29 (96.7%)	20 (95.2%)	9 (100%)	0.513^∞^	17 (94.4%)
1 = impaired, but able to carry out ADL without assistance	1 (3.3%)	1 (4.8%)	0 (0.0%)		1 (5.6%)
Antiplatelets *n* (%)					
0 = none	1 (3.3%)	1 (4.8%)	0 (0.0%)	0.887^∞^	0
1 = single agent	21 (70.0%)	14 (66.7%)	7 (77.8%)		12 (66.7%)
3 = dual therapy	8 (26.7%)	6 (26.8%)	2 (22.2%)		6 (33.3%)

*Note:* *p* values compare completers versus noncompleters.

Abbreviations: ABI, ankle‐brachial index; BMI, body mass index; HTN, hypertension; TBI, toe‐brachial index.

^∗^Independent sample *t*‐test.

^#^Fisher’s exact test.

^∞^Mann–Whitney *U*.

Baseline characteristics were broadly comparable between participants who completed the study and those who withdrew (Table 2). Participants who withdrew were slightly younger than completers (65.0 ± 8.7 vs. 71.3 ± 7.1 years; *p* = 0.046), and Rutherford classification differed between groups (*p* = 0.024). Completers tended to have more severe PAD, with 52.4% classified as Rutherford Grade 3, compared with 11.1% among noncompleters. No other significant differences were observed across demographic or clinical variables, including sex, body mass index, toe–brachial index, diabetes, smoking status, hypertension, functional status, cardiac or pulmonary comorbidity, hyperlipidaemia or antiplatelet use (all *p* > 0.05). There is no statistically significant difference in mean lowest ABI, although a trend towards a difference was observed (*p* = 0.057).

Overall, attrition was largely unrelated to baseline demographics or most measures of disease severity and comorbidity burden, supporting study feasibility despite dropout. Although small differences in age and Rutherford classification were observed, these do not suggest systematic bias in participant withdrawal.

### 8.2. Qualitative Findings

**Figure 3:**
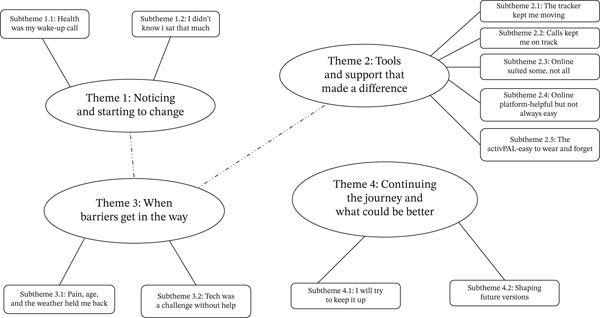
Thematic map illustrating relationships between themes and subthemes.

A group of 13 participants were interviewed (characteristics are shown in Table 3). The duration of the interviews ranged from 19 to 41 min. The average duration was 26 min. A total of four themes were developed. A thematic map of the themes and subthemes of the qualitative analysis is shown in Figure 3. Four overarching themes and 11 subthemes were developed through reflexive thematic analysis. These themes reflect participants’ evolving awareness and motivation, their engagement with various components of the programme and the barriers and facilitators influencing behaviour change (Appendix A).

**Table 3:** Characteristics of the 13 participants interviewed.

Participant ID	Gender	Age	Rutherford classification	BMI	Lowest ABI
1	F	69	2 moderate	32.3	0.88
2	M	67	3 severe	33	0.28
3	M	62	3 severe	26.5	0.65
4	F	74	3 severe	26.17	0.58
5	M	74	3 severe	27.5	0.55
6	F	69	2 moderate	23.5	0.8
10	M	72	2 moderate	30.6	1.2
11	M	69	3 severe	29	0.87
13	F	92	3 severe	22.3	0.42
15	M	71	3 severe	21.9	0.57
18	F	59	1 mild	38.7	0.63
20	F	80	3 severe	31.1	0.7
21	M	62	3 severe	28.4	0.75

#### 8.2.1. Theme 1

This theme explores participants’ initial motivations for taking part in the programme, their emerging awareness of sedentary behaviour and early attempts at change. Participants’ desire to improve health, curiosity and engagement with the programme led to increased recognition of sitting time and early shifts in behaviour. Two subthemes were developed through reflexive thematic analysis. First, ‘Health was my wake‐up call’ reflects participants’ motivation to improve health and manage pain. Second, ‘I didn’t know I sat that much’ captures new awareness of sedentary habits and their health impact.

##### 8.2.1.1. Subtheme 1.1: Health Was My Wake‐Up Call

Participants reported joining the programme to manage pain, improve activity and enhance well‐being.

Participants’ motivations for taking part in the programme were largely centred around a desire to improve their physical health and daily routines, particularly in relation to pain and inactivity. Many participants expected that the intervention might help alleviate physical discomfort, especially leg pain. Others expressed interest in using the programme to support lifestyle changes, become more active or break up sedentary patterns in their daily routines. For some, the decision to take part was also linked to curiosity about whether the intervention could positively influence their general well‐being. One participant shared, ‘I wanted to see if it would help me be more active in my daily routine and to change my lifestyle affected by depression and back pain’ (Participant 1, female, 69 years). Another reflected, ‘I thought it might have an impact on the pain I feel’ (Participant 2, male, 67 years).

##### 8.2.1.2. Subtheme 1.2: I Didn’t Know I Sat That Much

Many described a new awareness of how much they sit and its health implications, prompting greater reflection and attention to their routines.

Participants consistently reported that taking part in the programme increased their awareness of sedentary behaviour. Several described the experience as positive and eye‐opening, noting that it made them think more critically about how much time they spent sitting each day. Some participants reflected that the programme prompted them to pay more attention to their daily sitting habits, while others highlighted that the intervention helped them understand what constitutes sitting time and encouraged them to make changes. A few participants commented on how the information was new to them and increased their awareness of the potential health risks associated with prolonged sitting. ‘The information about sitting time was new to me. I hadn’t realised how much it could affect my health’ (Participant 4, female, 74 years). Others described becoming more conscious of their daily routines: ‘It made me think more about my sitting time’ (Participant 5, male, 74 years), and ‘This experience made me think more about how much I sit’ (Participant 10, male, 72 years).

#### 8.2.2. Theme 2

This theme focuses on participants’ experiences with the programme components, including coaching support, digital tools and online education. It reflects on perceived usefulness and the acceptability of remote delivery. Five subthemes were developed through reflexive thematic analysis. The tracker kept me moving captures how vibration prompts raised awareness and encouraged movement. Calls kept me on track reflects how weekly calls motivated and supported behaviour change. Online suited some, not all, describes how the online education was convenient for some but challenging for others. Feedback on the online education was mixed: Participants generally found it helpful, but some reported difficulties with usability. In contrast, the activPAL device was described as comfortable and easy to wear.

##### 8.2.2.1. Subtheme 2.1

The activity tracker (Huawei Band 6), particularly its vibration reminders, was widely described as a helpful and practical tool for increasing awareness of movement and sitting time. Participants valued how it encouraged them to break up long periods of inactivity by prompting them to move after 60 min of sitting.

One participant described the watch as ‘comfortable, easy to charge, and lasted long’, noting that ‘the vibrations reminded me to move and helped keep me aware of my activity’. Another participant shared that the tracker ‘helped a lot’ by drawing attention to both prolonged sitting and step count. A participant explained, ‘The tracker kept me aware of my sitting time. Although it sometimes failed to connect to my mobile, it was very useful, especially when it vibrated to remind me to move’ (male, 69 years). Similarly, another participant remarked, ‘It was effective, as the tracker reminded me to move’ (Participant 13, female, 92 years).

Others echoed these experiences. Some participants stated that the tracker helped them stay mindful of their movement throughout the day, particularly through step counts and vibration reminders. One participant said it ‘made a big difference’ to their activity, while Participant 10 appreciated its simplicity and daily use, stating it ‘encouraged me to sit less’.

##### 8.2.2.2. Subtheme 2.2: Calls Kept Me on Track

Participants consistently described the weekly coaching calls as a valuable and supportive component of the intervention. The calls served as regular reminders and a source of motivation, helping participants stay aware of their sitting habits and offering practical advice on how to break up prolonged sedentary periods. Several participants highlighted that the sessions effectively reinforced key messages, especially the importance of reducing and interrupting extended sitting time. Others emphasised the motivational benefit of the calls, describing them as helpful in maintaining focus and encouraging positive behavioural changes.

Overall, the coaching calls were viewed as an engaging, reassuring check‐in that supported both awareness and action towards reducing sedentary behaviour. As one participant put it: ‘The calls were really helpful focusing on sitting time and that was good’ (Participant 6, female, 69 years). Another added: ‘The calls were a good check‐in. The sessions motivated me to break up my sitting time and make changes’ (Participant 10, male, 72 years).

##### 8.2.2.3. Subtheme 2.3

Participants generally found the online education convenient and well‐suited to their needs, especially those living in remote areas, who appreciated not having to travel long distances. Many described it as easier or preferable to attending in person, with some stating it enabled them to complete the programme fully. As Participant 15 noted, ‘It was easier to access from home” (male, 71 years). While the convenience of online access was widely acknowledged, a few participants reported difficulties due to limited digital literacy or lack of technical support, particularly when using links or navigating the online education sessions (Participants 6, 10, 18 and 20). One participant shared, ‘It was handy, but I struggled a bit because I’m not very good with technology’ (Participant 18, female, 59 years).

##### 8.2.2.4. Subtheme 2.4

Participants’ experiences with the online component of the intervention were mixed, reflecting varying levels of digital literacy and comfort with technology. Some participants found the platform clear, informative and easy to navigate. For instance, one participant found the ‘top tips and animation helpful and easy to follow’ (Participant 3, male, 62 years) while another participant noted that the online sessions ‘provided useful tips to reduce sitting time’ and were ‘easy to understand’ (Participant 11, male, 69 years). A third participant acknowledged initial hesitation but eventually ‘got used to it’ and found the information ‘new and helpful’ (Participant 4, female, 74 years).

However, several participants encountered challenges related to technology use. Some expressed a lack of confidence or familiarity with online tools, which made it difficult to engage with the digital components independently. For example, Participant 10 stated, ‘I can’t use a computer’, while one participant noted that although they liked the graphics, they relied on their daughter for help support that ‘wasn’t always available’. Two other participants described the system as ‘hard to use’ and ‘difficult for someone not used to online tools’. Despite these barriers, a number of participants still valued the programme content. Participant 21 (male, 62 years) reflected, ‘It was difficult… but the information was helpful’. Similarly, Participant 1 (female, 69 years) shared, ‘Not easy to use or follow, but good overall. Learned more about my daily sitting’, while Participant 2 (male, 67 years) added, ‘Thought it might be tricky at first, but I got through it just fine’.

##### 8.2.2.5. Subtheme 2.5

Overall, participants found the activPAL device acceptable and easy to use, with straightforward instructions supporting independent use even among older adults. Several noted that they ‘barely noticed it after a while’ or that ‘it was fine to use’. The device caused minimal disruption to daily routines, with only occasional removal required for activities like swimming. ‘I barely noticed it after a while’ (Participant 5, male, 74 years). ‘It did not really change anything, and I did not have any problems with it’ (Participant 6, female, 69 years).

Clear instructions, including helpful photos (Participant 10) and hospital‐based fitting (Participants 11 and 15), supported correct placement and boosted user confidence. Importantly, no significant problems were reported. Minor issues, such as accidental water exposure, were managed appropriately thanks to prior guidance.

#### 8.2.3. Theme 3: When Barriers Get in the Way

This theme captures key barriers that affected participants’ full engagement with the programme such as physical limitations, low motivation, weather and technology‐related issues while also highlighting aspects influencing its overall feasibility, including the practicality of using digital tools and completing programme tasks in everyday life. Two subthemes were developed through reflexive thematic analysis. Barriers to Engagement: Pain, age, weather and limited digital skills affected some participants’ ability to engage fully.

##### 8.2.3.1. Subtheme 3.1

Pain, fatigue, weather and ageing affected participants’ ability to stay active and fully engage with the programme, raising important considerations for feasibility. Participants identified several personal and contextual barriers. As one participant (Participant 1, female, 69 years) explained, ‘My back pain and depression sometimes made it harder to stay active’. Similarly, another participant (Participant 4, female, 74 years) shared, ‘Sometimes moving around was difficult because of my pain’, while Participants 3 and 20 described how ‘The wintertime also worsened my leg pain. The weather made it difficult to stay active’.

Age and motivation also influenced engagement. One participant (Participant 15, male, 71 years) reflected, ‘From my point of view, I think age is a barrier and I lack urgency since I’ve too much time on hand’. Another added, ‘I sometimes struggled with dizziness, tiredness, and lack of motivation’ (Participant 18, female, 59 years).

##### 8.2.3.2. Subtheme 3.2

Some participants experienced difficulty navigating the online platform or using devices, often requiring support from family members or encountering usability issues. Several participants expressed a need for additional help when engaging with the online education component. One participant (Participant 10, male, 72 years) mentioned, ‘I can’t use a computer, so the online part was difficult for me to complete’.

While some participants found the content helpful and the programme generally clear, others encountered challenges due to limited familiarity with digital tools. Many relied on family members for assistance: ‘The online part was hard for me to use so I asked my daughter for help’ (Participant 13, female, 92 years). Some suggested that more guidance would benefit those less confident with technology, while others simply found the online education difficult to use.

A few participants also reported technical issues with the activity tracker, such as problems connecting it to their mobile phones. Nevertheless, they still found the device helpful. As one participant (Participant 11, male, 69 years) remarked, ‘I had some trouble connecting the tracker to my mobile. But it did vibrate when I sat for a long time’.

#### 8.2.4. Theme 4

This theme captures participants’ reflections on the impact of the programme, including their intentions to continue the strategies, perceived benefits and suggestions for improvement. It reflects the perceived acceptability and potential for promoting sustained behavioural change. Two subthemes were developed through reflexive thematic analysis. Participants expressed motivation to continue the behavioural changes and offered suggestions to enhance future versions, particularly around digital accessibility and device usability.

##### 8.2.4.1. Subtheme 4.1

Participants generally expressed a strong intention to continue using the behavioural strategies introduced during the programme. Many found them helpful in staying active and more aware of their sitting time. Some participants noted that they had already started integrating these strategies into their daily routines and had shared the information with others. While a few acknowledged that their routines had not changed significantly yet, they still expressed a willingness to persist with the strategies moving forward. Others highlighted plans to walk more regularly and remain focused on reducing sedentary time, reflecting a sustained motivation to apply what they had learned. ‘I’ll include the programme into my daily routine. Also, I’ve told my friends about it’ (Participant 2, male, 67 years) and ‘I plan to keep using them because they help me stay aware of my sitting time’ (Participant 3, male, 62 years).

##### 8.2.4.2. Subtheme 4.2

Participants offered a few suggestions to improve the programme, primarily focusing on technology‐related aspects. Some recommended making the online platform more accessible for individuals with limited digital skills, noting that simpler navigation and clearer instructions could enhance engagement. Improving the reliability of the activity tracker’s connection to mobile phones was also suggested. Additionally, one participant recommended offering alternative strap materials for the activity tracker, such as leather or metal, to reduce skin irritation. ‘It would be great to make sure to connect the watch to the phone’ (Participant 11, male, 69 years). ‘It would be good to make the online programme a bit easier for people who are not good with technology’ (Participant 18, female, 59 years).

**Figure 4:**
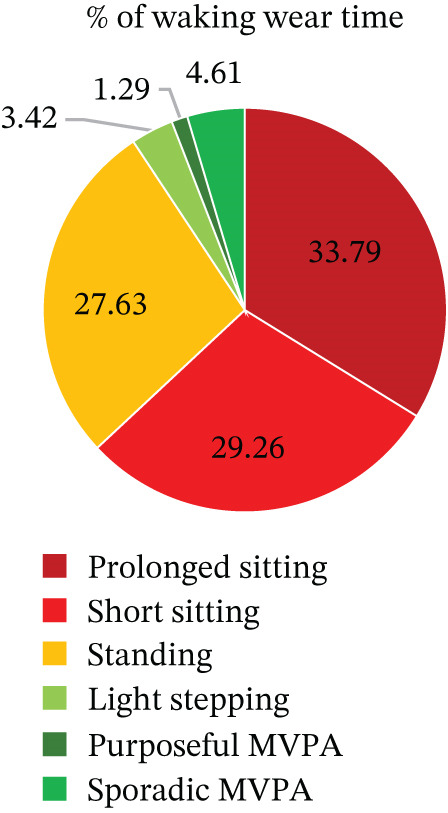
Baseline percentage of waking hours spent in sedentary and active behaviours (mean %).

**Figure 5:**
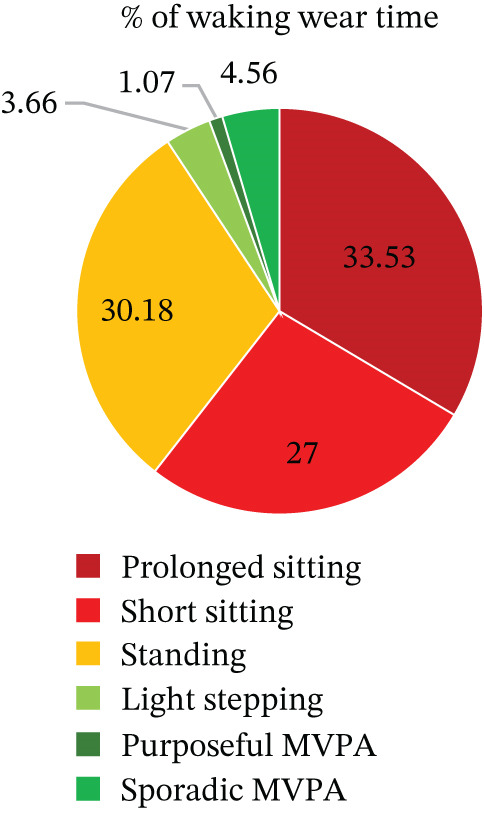
Postintervention (12 weeks) percentage of waking hours spent in sedentary and active behaviours (mean %).

Eighteen of the 21 participants provided valid pre‐ and postmeasurements. Figures 4 and 5 show the percentage of waking hours spent in sedentary and active behaviours at baseline and at 12 weeks postintervention for participants with complete pre‐ and postintervention activPAL data (*n* = 18).

### 8.3. Sedentary Time and Physical Activity

The descriptive statistics for the activPAL variables (*n* = 18) are shown in Table 4, and Figures 4 and 5 show the percentage of waking hours spent in sedentary and active behaviours at baseline (preintervention) and at 12 weeks (postintervention), respectively. At baseline, participants spent 63% of their waking day sitting (9.58 h/day), which was slightly lower at 12 weeks (60%). Data suggest that prolonged sitting time reduced, standing time increased and stepping time remained similar across time points.

**Table 4:** Descriptive statistics for the activPAL variables (*n* = 18).

Average hours/day variables	Baseline	12 weeks	Absolute change	% change
Valid waking wear time^a^	15.20 (±1.53)	14.66 (±1.51)	−0.54 h	−3.55%
Total sitting time^a^	9.58 (±1.88)	8.88 (±2.87)	−0.70 h	−7.31%
Total short sitting bout time^a^	4.45 (±1.50)	3.96 (±1.41)	−0.49 h	−11.01%
Total prolonged sitting time bouts^a^	5.13 (±2.02)	4.91 (±2.32)	−0.22 h	−4.29%
Total light stepping time (step cadence < 100 steps/min)^b^	0.48 (0.32–0.65)	0.49 (0.32–0.74)	+0.01 h	+2.08%
Total MVPA stepping time^b^ (step cadence > 100 steps/min)	0.61 (0.41–1.15)	0.58 (0.40–1.11)	−0.03 h	−4.92%
Purposeful MVPA time^b^ (MVPA lasting ≥ 1 min)	0.06 (0.016–0.297)	0.016 (0.002–0.213)	−0.04 h	−70.91%
*Proportions meeting recommendations*				
Achieving ≥ 150 min/week MVPA (%)	88.88%	83.33%	−5.55%	−6.27%
Achieving < 9.5 h total sitting/day (%)	44.44%	66.67%	+22.23%	+50.06%

^a^Mean (±SD).

^b^Median (IQR).

### 8.4. 6MWT Results

Walking capacity was assessed using the 6MWT via the Timed Walk application at baseline and again after the 12‐week intervention. Due to the temporary unavailability of the 6MWT app on Android devices, some participants were unable to complete this outcome measure. Consequently, complete 6MWT data were available for 14 participants. The mean 6MWT distance at baseline was 334.21 m (SD = 112.41), while the mean postintervention distance was 329.39 m (SD = 95.21). This represents a mean reduction of 4.82 m over 12 weeks, corresponding to a 1.44% decrease in walking distance. While the change in 6MWT distance over 12 weeks was minimal and may reflect individual variability, it is notable that overall walking capacity remained relatively stable throughout the intervention period.

## 9. Discussion

The findings of this study demonstrate the feasibility of delivering a remotely delivered intervention to reduce and break up sedentary time in people with PAD. Of the 76 individuals approached, 30 consented to take part, and 21 (70%) completed the study. Eighteen participants completed both baseline and 12‐week follow‐up activPAL measurements. Recruitment of patients with PAD into clinical trials is often difficult due to comorbidities, low engagement and logistical barriers associated with their health condition [22, 23]. Only around half of clinical studies in this field achieve their target sample size and just as many do so within the intended timeframe [24].

Recruitment and retention rates, though reflecting known challenges in PAD populations, were sufficient to support feasibility testing. Participants found the intervention acceptable, valuing wearable prompts, online education and supportive coaching calls. Remote delivery likely aided retention by reducing travel and time burdens for people with claudication, consistent with findings from Brierley et al. [25].

In the present study, attrition rate from consent was 30%, which is relatively high for a short follow‐up period and indicates the need for strategies to improve retention in future work. Nevertheless, recruitment, data completion and qualitative findings were consistent with feasibility objectives, suggesting that the approach may be suitable for refinement and evaluation in a larger randomised controlled trial (RCT).

Engagement with the intervention components shows that people with PAD can participate in remotely delivered behaviour change strategies. Most participants completed 9–12 of the 12 scheduled coaching calls, with missed calls successfully rescheduled, demonstrating the value of a flexible approach. Consistent use of online education modules and wearable prompts suggests that technology‐based components can be integrated into daily routines. Occasional support from family members was needed to navigate digital tools, highlighting the importance of addressing digital literacy and providing guidance for those less familiar with technology. In line with evidence from other clinical groups, blended interventions combining technology‐based self‐monitoring with human support seem more effective for sustaining behaviour change than technology alone [25, 26].

### 9.1. Feasibility and Acceptability of the Intervention

Adherence to the intervention was generally good, with most participants actively using the physical activity tracker and engaging in weekly coaching calls. Digital health interventions offer promise for improving care in older adults but can present usability challenges, particularly for individuals unfamiliar with technology [27]. Issues such as poor design, small fonts and complex navigation may limit engagement [28]. In this study, assistance from family members was required a few times, but overall engagement indicated that the intervention was both feasible and acceptable. This aligns with findings by Mercer et al. [29], who reported that older adults with chronic illness generally find wearable physical activity devices useful and acceptable, though some may require support with setup and data interpretation.

The current study adopted the same online education session from the SMART Work and Life study [19] to deliver key sedentary behaviour messages. Consistent with SMART Work and Life, participants found the session informative and acceptable. Perceived accountability clearly played a role in participants’ engagement and behaviour change. The weekly coaching calls served not only as a source of motivation and support but also as a consistent point of contact that helped participants stay focused and on track. Several participants described the calls as a helpful ‘check‐in’ that made them more aware of their sitting habits and encouraged them to act. For example, one participant stated that the sessions ‘motivated me to break up my sitting time and make changes’, while another participant said the calls were ‘really helpful focusing on sitting time’. Consistent with the RESIT study, participants in the RESIT intervention reported that health coach support was one of the most valuable aspects of the programme. The regular coaching sessions were viewed as acceptable and supportive, helping to sustain motivation and adherence [30].

This suggests that knowing someone would follow up and discuss their progress created a sense of responsibility, which likely enhanced adherence to the programme and reinforced key behaviour change messages. McFeeley et al. [31] similarly reported that coaching delivered through digital health programmes supports the adoption and maintenance of healthy lifestyle behaviours.

### 9.2. Sedentary Behaviour and Physical Activity

People with PAD are typically highly sedentary and engage in lower levels of physical activity compared with the general population [3]. Participants in the current study spent 9.6 h per day (576 min/day) sedentary, which is aligned with previous research indicating 433–640 min/day of sitting [32–34]. This is higher than observed in older adults without PAD [35], reflecting the mobility limitations and discomfort associated with PAD.

The findings of this study suggest that the intervention has the potential to reduce sitting time, although observed differences at follow‐up were modest (~3% of waking time). These changes were primarily due to reductions in prolonged sitting, accompanied by increases in standing time. Notably, the proportion of participants achieving < 9.5 h per day of sitting [6] increased from 44.4% preintervention to 66.7% postintervention, representing a 22.2% absolute and 50% relative improvement. Similarly, Perks et al. [30] reported reductions in sitting time of 0.9 h/day following an 8‐week personalised activity plan to reduce sitting time in people with IC, supporting the potential of targeted interventions in this population [26, 36].

These findings align with previous interventions in older adults, which generally reduced total sedentary time, particularly when multiple strategies such as education, self‐monitoring and goal setting were included [37, 38]. Evidence from PAD‐specific interventions remains limited. A systematic review [3] highlighted a few intervention studies in this population. Among them, Laslovich et al. [36] reported significant reductions in sedentary time using a 12‐week home‐based intervention with self‐monitoring, goal setting and personalised feedback, whereas Whipple et al. [39], using supervised exercise therapy, found no overall reduction and considerable individual variability, including increases in sedentary behaviour. These findings suggest that exercise alone may be insufficient to reduce sitting time in PAD. Interventions that explicitly target sedentary behaviour using behavioural strategies, as employed in the current study, may be necessary to achieve meaningful changes in sedentary time.

Despite other positive behavioural changes (e.g., less sitting and more standing), participants’ engagement in moderate‐to‐vigorous activity showed little change, likely reflecting the physical limitations common in people with PAD or IC, who often find it challenging to sustain faster walking cadences. Consistent with findings by Perks et al. [26], individuals with IC tend to maintain moderate‐intensity activity only for brief periods. Furthermore, as existing accelerometer cut points are based on healthy populations, they may overestimate actual intensity levels in PAD. Lower accelerations and step cadences may correspond to greater exertion, as seen in other cardiovascular and older populations. This underscores the need to develop PAD‐specific thresholds for activity intensity. The near absence of vigorous‐intensity activity further illustrates the difficulty people with IC experience in reaching higher exertion levels [26].

Integration of quantitative and qualitative findings provides context for feasibility outcomes. Participants reported that the activity tracker, with its vibration prompts, and weekly coaching calls were motivating and increased awareness of sedentary behaviour, supporting engagement and likely contributing to the high recruitment rate. However, barriers such as pain, fatigue, age‐related limitations, weather and variable digital literacy affected participants’ ability to fully engage and may explain some attrition and incomplete activPAL data. Suggestions from participants including clearer online instructions, improved tracker connectivity and alternative strap options highlight practical ways to enhance adherence and device usability in future studies.

### 9.3. Functional Outcomes and the 6MWT

Functional walking ability, assessed via the remote 6MWT [13], showed a small decline of 4.82 m (−1.44%) over 12 weeks period. The decline is below the minimal clinically important difference (8–20 m) for PAD [40]. Walking performance remained stable, indicating that although sedentary time was reduced, the increase in light‐intensity activity was not sufficient to enhance functional capacity. Previous meta‐analyses of home‐based exercise programmes in PAD have demonstrated clear benefits for pain‐free and maximal walking distance [41]. These findings highlight that interventions which specifically target walking exercise may be more effective at improving functional outcomes, whereas strategies that focus primarily on reducing sedentary behaviour may help maintain but not enhance walking capacity.

### 9.4. Implication of Findings

Participant feedback indicates that the intervention raised awareness of sedentary behaviour and its potential impact on health, prompting reflection and behaviour change. Wearable trackers, such as the Huawei Band 6, were generally well‐received and served as useful prompts for movement, helping participants monitor sitting time and increase activity. However, occasional technical issues highlight the importance of ensuring device reliability and providing clear guidance on their use.

Weekly coaching calls emerged as a highly valued component, offering motivational and practical support that reinforced behaviour change messages and helped participants stay on track. These personalised check‐ins enhanced engagement and encouraged sustained reductions in sedentary time.

The online delivery was largely appreciated for its convenience, particularly by participants in rural or remote areas, yet also revealed notable barriers for those with limited digital literacy. Some struggled to use links or navigate the online sessions without external help. These challenges underscore the need for digital health interventions to include tailored technical support, user‐friendly platforms and accessible content. For older adults or participants unfamiliar with technology, offering clear instructions, alternative delivery formats or additional support options could improve both feasibility and equity of access.

No adverse events were reported, and participant feedback highlighted the value of reminders to reduce prolonged sitting. Future interventions may benefit from enhanced personalisation of behaviour change goals, integration of tailored activity plans and flexible support models that account for varying levels of technological literacy and health status. Incorporating strategies such as motivational interviewing or problem‐solving may further support engagement and encourage more sustained increases in structured activity.

Overall, these findings emphasise the importance of incorporating flexible, supportive and inclusive approaches in digital health interventions to ensure that they are accessible and acceptable to a wide range of users.

## 10. Conclusion

This study demonstrated that a 12‐week, remotely delivered intervention for people with PAD, incorporating activity trackers, online education and coaching calls, was both feasible and acceptable. Participants reported the intervention as informative and viewed it positively. Recruitment targets were met, though strategies to improve retention and activPAL compliance would be needed for a larger trial. The intervention showed potential to reduce sitting time. Overall, these findings offer valuable insights into recruitment, retention and measurement procedures and will help inform the design of a future adequately powered RCT to evaluate effectiveness.

### 10.1. Implications for Future Trials: Integration of Quantitative and Qualitative Findings

Triangulation of quantitative and qualitative findings provides a comprehensive understanding of intervention feasibility and informs strategies for refinement. Quantitatively, recruitment was strong, while retention and activPAL data completeness were slightly below commonly referenced thresholds. Qualitative interviews revealed that participants valued coaching calls and wearable devices, but practical barriers such as acute illness, physical limitations, environmental factors and challenges with digital literacy affected engagement and device wear. Integrating these data allows identification of the behavioural and contextual factors influencing feasibility and highlights where improvements are needed.

Based on this integrated insight, future trials could incorporate targeted strategies to enhance adherence and data completeness. These may include automated reminders, active monitoring of device wear, personalised feedback, simplified and more accessible digital materials, enhanced technical support and motivational approaches such as appropriate incentives. These measures are likely to improve retention, device compliance and overall study feasibility in a future RCT.

## Funding

This research is supported by the National Institute for Health and Care Research (NIHR) Leicester Biomedical Research Centre (BRC).

## Conflicts of Interest

The authors declare no conflicts of interest.

## Data Availability

The datasets analysed during the current study are available from the corresponding author upon reasonable request. Data will be provided in anonymised form and in accordance with the study′s ethical approval.

## Appendix A

**Table A1:** Themes and illustrative quotes for the qualitative analysis.

**Theme and subtheme**	**Illustrative quotes**
Theme 1: Noticing and Starting to Change	
Subtheme 1.1: Health Was My Wake‐Up Call	● I wanted to see if it would help me be more active in my daily routine and to change my lifestyle affected by depression and backpain (Participant 1).
	● I thought it might have an impact on the pain I feel (Participant 2).
	● I wanted to know if it would help with my leg pain (Participant 3).
	● I was curious to see if it would help with the pain I feel (Participant 4).
	● I wanted to see if it could improve my daily routine (Participant 5).
	● I was looking to see if it’d make me feel any better (Participant 6).
	● I just wanted to see if it could help me switch up my daily routine and be a bit more active (Participant 10).
	● Hoping it might help with the pain in my leg (Participant 11).
	● I thought it might help with the pain (Participant 13).
	● To find ways to be more active (Participant 15).
	● I was looking to move more and cut down on sitting during work (Participant 18).
Subtheme 1.2: I Didn’t Know I Sat That Much	● It made me think more about how much time I spend sitting (Participant 1).
	● It made me aware of the time I spend sitting and what counts as sitting time (Participant 2).
	● The information about sitting time was new to me. I had not realised how much it could affect my health (Participant 4).
	● It made me think more about my sitting time (Participant 5).
	● This experience made me think more about how much I sit (Participant 10).
	● This programme made me more conscious of my sitting time and encouraged me to change it (Participant 11).
	● Taking part made me think more about how much time I spend sitting (Participant 13).
	● Taking part was useful in making me more aware of my sitting time and the importance of moving more (Participant 15).
	● This programme made me more aware of how much time I spend sitting and how it affects my health (Participant 20).
	● Taking part helped me become more aware of the problems caused by sitting too much (Participant 21).

Theme 2: Tools and Support That Made a Difference	
Subtheme 2.1: The Tracker Kept Me Moving	● Watch was comfortable, easy to charge, and lasted long. Vibrations reminded me to move. Tracker kept me aware of activity (Participant 1).
	● The tracker helped a lot. It vibrated when I sat too long and helped me notice how many steps I was taking (Participant 2).
	● The tracker reminded me to move more (Participant 3).
	● It vibrated when I had been sitting too long, which helped me stay aware of my movement (Participant 4).
	● The tracker kept me aware of my activity (Participant 5).
	● The watch made a big difference in my activity (Participant 6).
	● The tracker was simple and encouraged me to sit less. I used it every day and it was very useful (Participant 10).
	● The tracker kept me aware of my sitting time. Although it sometimes failed to connect to my mobile, it was very useful, especially when it vibrated to remind me to move (Participant 11).
	● It was effective, as the tracker reminded me to move (Participant 13).
	● The tracker was useful as a reminder, and the weekly calls helped reinforce the importance of moving more. The tracker did not always connect to my phone, which was frustrating (Participant 15).
	● The tracker was a helpful reminder to keep moving (Participant 21).
Subtheme 2.2: Calls Kept Me on Track	● It reminded me about sitting, how to reduce my sitting time and other helpful tips (Participant 2).
	● The calls were great reminders which helped me stay aware of my movement (Participant 4).
	● They focused on important things like how to sit less (Participant 5).
	● The calls were really helpful focusing on sitting time and that was good (Participant 6).
	● The calls were a good check‐in. The sessions motivated me to break up my sitting time and make changes (Participant 10).
	● They focused on useful things like how to decrease sitting (Participant 11).
	● I think it was effective as the calls kept me on track, and they covered important things like reducing and breaking up sitting time (Participant 13).
	● The weekly calls helped support the importance of moving more (Participant 15).
	● The weekly sessions helped keep me motivated and focused on ways to reduce sitting time, which I found helpful (Participant 18).
	● Useful as they focused on reducing sitting time and finding ways to reduce it (Participant 20).
Subtheme 2.3: Online Suited Some, Not All	● It was easier for me because I live in Donegal. I do not think I would have joined if it was all in the hospital because the travel would have taken too long. I’d recommend it to other patients (Participant 2).
	● Online worked fine for me more than in person (Participant 3).
	● The online option was perfect for me (Participant 4).
	● I would have liked more help with using it (Participant 6).
	● Online worked fine for me but I found it difficult to use the link because I do not use a computer (Participant 10).
	● Online was better than face to face for me and I completed all of it (Participant 11).
	● It was easier to access from home (Participant 15).
	● It was handy, but I struggled a bit because I’m not very good with technology (Participant 18).
	● Being online was helpful, but I found the online education part difficult to use (Participant 20).
	● The online access worked well for me (Participant 21).
Subtheme 2.4: Online Platform—Helpful but Not Always Easy	● Not easy to use or follow, but good overall. Learned more about my daily sitting (Participant 1).
	● Thought it might be tricky at first, but I got through it just fine (Participant 2).
	● Top tips and animation were helpful and explained things well. I found it easy to follow and understand (Participant 3).
	● At first, I wasn’t sure how it would go, but I got used to it. The info about sitting was new to me and helpful. It was easy to follow (Participant 4).
	● It wasn’t easy to use, so I waited for my daughter to help. I was surprised to learn what counts as sitting. It was clear and easy to understand once I got help (Participant 5).
	● It was difficult because no one was there to help me (Participant 6).
	● I cannot use a computer, so the online part was difficult for me to complete (Participant 10).
	● I completed all the online sessions. The link provided useful tips to reduce sitting time, and the information was easy to understand (Participant 11).
	● It was not easy for me because I’m not used to using devices, so I needed my daughter’s help. She wasn’t always available, but I liked the graphics (Participant 13).
	● It was useful. I did all the online part. The animations helped explain things. I did not fill the worksheet—some bits felt the same. Maybe make the messages clearer. It was easy to use. Some parts could explain better how to move more at home (Participant 15).
	● It was useful, but I found it hard to use because I’m not good with technology (Participant 18).
	● I found the online part hard to use and difficult for someone like me who’s not used to online tools (Participant 20).
	● It was difficult for me to use, but the information was helpful (Participant 21).
Subtheme 2.5: The activPAL—Easy to Wear and Forget	● It did not affect my routine much; I just took it off for swimming and used the extra patch after (Participant 2).
	● It was fine to use, and I had no issues with it (Participant 3).
	● It did not change anything, and I did not face any problems (Participant 4).
	● I followed the instructions, and it was fine. I barely noticed it after a while (Participant 5).
	● It did not really change anything, and I did not have any problems with it (Participant 6).
	● It was fine to use, and I had no problems with its use as I’d the instructions with a photo (Participant 10).
	● It was fine to use as I got it on at the hospital by the doctor (Participant 11).
	● “I followed the instructions, and it was fine. One day, water spilled on it, but I remembered it was safe to wear in the shower, just not in the bath (Participant 13).
	● The device was fitted at the hospital, and everything was fine (Participant 15).

Theme 3: When Barriers Get in the Way	
Subtheme 3.1: Pain, Age and the Weather Held Me Back	● My back pain and depression sometimes made it harder to stay active (Participant 1).
	● The wintertime also my leg pain (Participant 3). The weather made it difficult to stay active (Participant 20).
	● Sometimes moving around was difficult because of my pain but I still managed to complete everything (Participant 4).
	● I cannot use a computer, so the online part was difficult for me to complete (Participant 10).
	● I had some trouble connecting the tracker to my mobile. But it was vibrating if I sit for long time (Participant 11).
	● The online part was hard for me to use so I asked my daughter for help (Participant 13).
	● From my point of view, I think age is a barrier and lack of urgency as I’ve too much time in hand (Participant 15).
	● I sometimes struggled with dizziness, tiredness, and lack of motivation (Participant 18).
Subtheme 3.2: Tech Was a Challenge Without Help	● The online education was not easy to use so I waited for my daughter to help me (Participant 5).
	● The online education was helpful, but I found some parts hard to use on my own and I think more guidance is needed for people who find online tools hard to use (Participant 6).
	● To be honest I found the online education part was hard (Participant 10).
	● The online part was hard for me to use so I asked my daughter for help (Participant 13).
	● I liked the content, but I struggled a little with the online education (Participant 18).
	● I found the online part difficult to use (Participant 20).
	● The programme was clear and easy to follow (Participant 21).

Theme 4: Continuing the Journey and What Could be Better	
Subtheme 4.1: I Will Try to Keep it Up	● I’ll keep using them because they help me stay active (Participant 1).
	● I’ll include the programme into my daily routine. Also, I’ve told my friends about it (Participant 2).
	● I plan to keep using them because they help me stay aware of my sitting time (Participant 3).
	● I plan to keep using them, and I’ve also shared what I learned with other people (Participant 4).
	● I’ll definitely keep using them (Participant 5).
	● I will try to keep using them, but my routine has not changed much yet (Participant 15).
	● Sure, I will continue trying to be more active and reduce my sitting time (Participant 18).
	● I plan to continue making changes and walking more regularly (Participant 20).
	● Yes, I plan to continue using these strategies to reduce my sitting time (Participant 21).
Subtheme 4.2: Shaping Future Versions	● It would be great to make sure to connect the watch to the phone (Participant 11).
	● It would be good to make the online programme a bit easier for people who are not good with technology (Participant 18).
	● Making the online platform easier to use would be helpful (Participant 20).
	● It would be good if the activity tracker had a leather or metal strap to avoid skin reactions (Participant 21).
